# Dung beetles from two sustainable-use protected forests in the Brazilian Amazon

**DOI:** 10.3897/BDJ.11.e96101

**Published:** 2023-03-16

**Authors:** Edrielly C. Carvalho, Maria Eduarda Maldaner, Vinicius Costa-Silva, Heivanice Sehn, Carol Franquini, Vinicius O. Campos, Vinicius P. Seba, Laís F. Maia, Fernando Z. Vaz-de-Mello, Filipe Machado França

**Affiliations:** 1 Departamento de Biologia e Zoologia, Instituto de Biociências, Universidade Federal de Mato Grosso. Laboratório de Scarabaeoidologia. Instituto de Biociências - UFMT, Cuiabá, Brazil Departamento de Biologia e Zoologia, Instituto de Biociências, Universidade Federal de Mato Grosso. Laboratório de Scarabaeoidologia. Instituto de Biociências - UFMT Cuiabá Brazil; 2 Programa de Pós-Graduação em Entomologia. Instituto Nacional de Pesquisas da Amazônia – INPA, Manaus, Brazil Programa de Pós-Graduação em Entomologia. Instituto Nacional de Pesquisas da Amazônia – INPA Manaus Brazil; 3 Programa de Pós-Graduação em Ecologia e Conservação da Biodiversidade (PPGECB), Universidade Federal de Mato Grosso - UFMT, Cuiaba, Brazil Programa de Pós-Graduação em Ecologia e Conservação da Biodiversidade (PPGECB), Universidade Federal de Mato Grosso - UFMT Cuiaba Brazil; 4 Laboratory of Integrative Entomology, Department of Animal Biology, Institute of Biology, University of Campinas, Campinas, Brazil Laboratory of Integrative Entomology, Department of Animal Biology, Institute of Biology, University of Campinas Campinas Brazil; 5 Programa de Pós-Graduação em Zoologia. Instituto de Biociências, Universidade Federal de Mato Grosso - UFMT, Cuiabá, Brazil Programa de Pós-Graduação em Zoologia. Instituto de Biociências, Universidade Federal de Mato Grosso - UFMT Cuiabá Brazil; 6 School of Biological Sciences, University of Bristol, Queens Road, BS8 1QU, UK, Bristol, United Kingdom School of Biological Sciences, University of Bristol, Queens Road, BS8 1QU, UK Bristol United Kingdom; 7 Programa de Pós-Graduação em Ecologia (PPGECO), Universidade Federal do Pará, Belém, PA, 66075-110, Brazil, Belém, Brazil Programa de Pós-Graduação em Ecologia (PPGECO), Universidade Federal do Pará, Belém, PA, 66075-110, Brazil Belém Brazil

**Keywords:** Amazonia, biodiversity, Coleoptera, dung beetles, Scarabaeinae, sustainable-use forests, tropical ecosystems

## Abstract

**Background:**

The Amazon Forest is one of the world's most biodiverse ecosystems and yet its protected areas are understudied concerning insects and other invertebrates. These organisms are essential for tropical forests due to their ecological processes, with some species being very sensitive to habitat disturbances. Dung beetles (Coleoptera, Scarabaeidae, Scarabaeinae) have been used as bioindicators for more than 30 years and were surveyed to assess the insect biodiversity of two sustainable-use forest reserves in the Brazilian Amazon.

**New information:**

We report inventories of dung beetles from two Amazonian forest reserves in Pará State, Brazil: the Tapajós National Forest and the Carajás National Forest. Surveys were carried out with baited-pitfall traps installed in 2010, 2016, 2017 and 2019. We collected a total of 3,772 individuals from 19 genera and 96 species. We highlight the importance of Amazonian protected areas as refugia for insect biodiversity, particularly dung beetles, which contribute to many key ecosystem processes.

## Introduction

The Amazon Forest has global importance for biodiversity, being amongst the world’s most diverse tropical ecosystems (Barlow 2018). The region's warm and humid climate makes Amazonian forests a unique ecosystem with extraordinary biodiversity levels, particularly for insects (Fearnside 2008, Sobral-Souza and Lima-Ribeiro 2017).

With the human footprint and climate extremes increasing within tropical regions (França et al. 2020a), protected areas are increasingly becoming the final refuges for biodiversity, including many restricted-range and highly threatened species (Rylands and Brandon 2005, Sollmann et al. 2008). Until 2009, around 54% of the remaining Amazon Forest in Brazil was part of a protected area network, ranging from strictly protected areas and indigenous lands to sustainable use forests (Soares-Filho et al. 2010). The importance of these protected areas goes beyond biodiversity conservation by sustaining local livelihoods (Naughton-Treves et al. 2005, Spinola et al. 2020), preventing climate-tipping points (Walker et al. 2009) and supporting the mitigation of climate changes through carbon accumulation and reduction of Greenhouse Gases (GHG) emissions from deforestation (Soares-Filho et al. 2010, Walker et al. 2020).

Despite the unparalleled contribution of insects to the totality of biodiversity (Zhang 2011) and their important role in many critical ecological functions (Nichols et al. 2008, Campbell et al. 2012, Dangles and Casas 2019), insects are understudied when compared to vertebrates (Dornelas and Daskalova 2020). For example, only 1.67% of the known invertebrate species have been assessed by the IUCN Red List of threatened species compared with 68.9% of all vertebrates (Kitching et al. 2020). The lack of studies with insects and invertebrates is also evidenced within Amazonian protected areas.

Dung beetles (Coleoptera, Scarabaeinae) are a key group of detritivore insects frequently used in ecological research linking biodiversity and ecosystem functioning (França et al. 2018, Griffiths et al. 2015). Through feeding and nesting in mammal dung, carrion or rotten fruits, dung beetles play important roles in nutrient cycling and other ecosystem functions (Halffter and Matthews 1966, Nichols et al. 2008). Given their quick responses to environmental degradation by anthropogenic and climatic disturbances (e.g. França et al. 2020a, França et al. 2020b), since the 1990s, dung beetles have been used as an efficient indicator of environmental quality in tropical forests (Halffter and Favila 1993, Davis et al. 2004, Larsen and Forsyth 2005, Spector 2006, Nichols et al. 2007, Gardner et al. 2008, Nichols et al. 2008, Culot et al. 2013). In general, anthropogenic activities lead to changes in dung beetle 'fitness' (through physiological stress: for example, França et al. (2016), Salomao et al. (2018)), species richness and abundance (Klein and Bert 1989, Halffter and Arellano 2002, Escobar et al. 2007).

Here, we: (1) present a list of dung beetle species surveyed at two sustainable-use protected forests in the Brazilian Amazon – the Tapajós National Forest and the Carajás National Forest (FLONAS); and (2) discuss insights associated with the species distribution and previous recordings in literature.

## Materials and methods

### Study region

**Figure 1.**
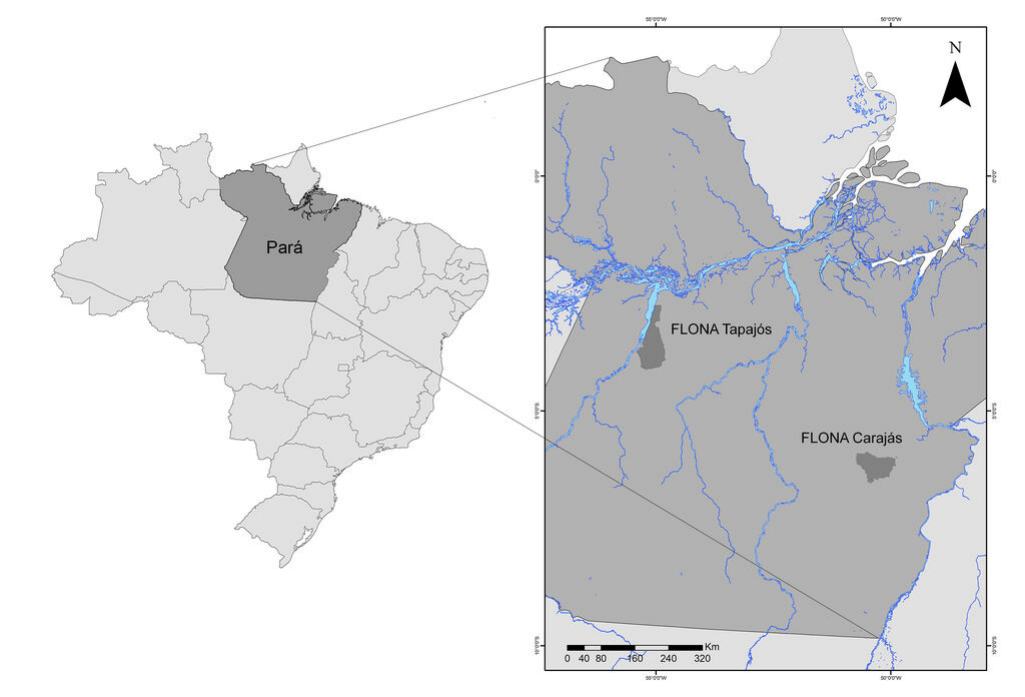
Map showing the localities of Tapajós National Forest (FLONA Tapajós) and Carajás National Forest (FLONA Carajás; both in dark grey) in Pará State (medium grey), in the north of Brazil (light grey).

The Tapajós National Forest and Carajás National Forest (hereafter 'Tapajós' and 'Carajás', respectively) cover 527,319 ha and 411,948 ha of Amazonian forests, respectively, spread across multiple municipalities in the State of Pará, Brazil (Fig. 1). These FLONAS are located in two Amazonian biogeographical regions with distinct socio-environmental contexts. Specifically, the Tapajós region has a more recent history of agriculture expansion and lower deforestation rates than the Carajás region (Braz 2016), with FLONA Carajás located within a mosaic of federal and state forest reserves, national parks and indigenous protected lands (Piló et al. 2015). The climate is characterised as hot-humid (Köppen's classification) and the annual average temperature is 25-26°C in both regions, which have short dry seasons in August-November (average precipitation [mm]: Tapajós = 1405.8) and July-September (Xingu = 84.8). Surveyed sites are within the 'terra-firm' forests, with vegetation varying depending on soil and relief.

### Sampling design

Dung beetles were sampled within a total of 13 forest sites (Carajás = 3 and Tapajós = 10). We surveyed Carajás in February–March 2019, while Tapajós forests were surveyed in June-July 2010, June–July 2016, March–April 2017 and Feb–March 2019. These field sites are part of the Long-Term Ecological Research Program of the Sustainable Amazon Network (PELD-RAS). At each of our forest sites, dung beetles were sampled at three sampling points (0, 150 and 300 m) along a 300-m transect. As in França et al. (2020b), we used three dung-baited pitfall traps arranged at the ends of a 2-m equilateral triangle at each sampling point, resulting in a total of 117 traps (21 and 96 pitfalls in Carajás and Tapajós, respectively). Pitfall traps were 1-litre plastic containers (14 cm in diameter; 9 cm deep) buried in the ground with the opening at ground level and protected from rain with a plastic lid suspended 15 cm above the surface. Each trap was part-filled with a saline killing solution, had a bait container with 35 g of dung (4:1 pig to human ratio, following Marsh et al. (2013)) supported by a wire above the trap and was left in the field for 48 hours.

## Data resources

All trapped dung beetles were collected and taken to the laboratory, where they were sorted, mounted and identified to species (using identification key or descriptions) or morphospecies. Voucher specimens were deposited at the Entomological Section of the Zoological Collection (CEMT) at the Federal University of Mato Grosso, Brazil (UFMT). Specimens were photographed using the Leica M250C Photomontage Equipment (UFMT/Finep) and an Olympus SZX16 stereomicroscope with expandable stream motion imaging software v. 2.5 (UoB/Liv Sidse Hansen Foundation). The morphospecies identification numbers are not indicating the amount of species collected at the sites and are purely reference numbers for species across multiple projects. The abbreviations *aff.*, *cf.* and *gp.* are qualifiers used in taxonomy to indicate different degrees of uncertainty of identification. The use of *aff.* and *cf.* follows Lucas (2012) and *gp.* indicates species group affinity.

The map showing the localities of Tapajós National Forest and Carajás National Forest was prepared using ArcGIS 10.8 software. Dung beetle data can be found at http://www.gbif.org/tools/data-validator/f1e2a538-5fea-4258-9b0e-27805b684404 (GBIF 2022).

## Checklists

### List and abundance of species present in FLONA Tapajós and Carajás

#### Scarabaeinae

Latreille, 1802

E17C7C59-4111-53D0-A7A5-BC0DDB7ABA8F

##### Notes

We collected 3,772 dung beetles from 96 species and 19 genera. Only 14 of the 96 identified species were found in both FLONAS (Table 1). *Canthidiumdeyrollei* was the most abundant species, with exclusive records from FLONA Tapajós. The three most diverse genera comprise distinct functional strategies in dung beetles: *Eurysternus* (endocoprids – i.e. residents in the dung resource); *Dichotomius* (paracoprids – i.e. tunnellers, where all species mostly dig tunnels close to or immediately below the resource) and *Canthon* (telecoprids – i.e. rollers), which could be an indicator of whole exploitation of dung resources in Amazonian forests. However, if abundance is considered, small paracoprids (especially *Onthophagus* species) were the most abundant functional group, which is expected for the Amazon Region (FVM, pers. obs). We discuss below the current knowledge about the distribution and ecology of each identified genera.

#### Anomiopus

Westwood, 1842

BD7AC088-1EE4-5A5A-9633-BB8469FE4D38

##### Notes

*Anomiopus* is a Neotropical genus with most species occurring in South America. The latest revision has 48 described species (Canhedo 2004, Canhedo 2006). Most *Anomiopus* species are collected with flight interception traps (FIT), Malaise traps, pitfalls baited with human dung (Canhedo 2006), light traps and bird faeces (Martinez 1959). Some species were observed in the Colombian Amazon landing on leaves during the day (Canhedo 2006). In our study, we found five species: Anomiopusaff.pereirari, *Anomipus* sp. 2, *Anomiopus* sp. 3, *Anomiopus* sp. 4 and *Anomiopus* sp. 5.

#### Ateuchus

Weber, 1801

823D3FBD-2B75-588A-8C55-15DEC4C58DCB

##### Notes

**Figure 2.**
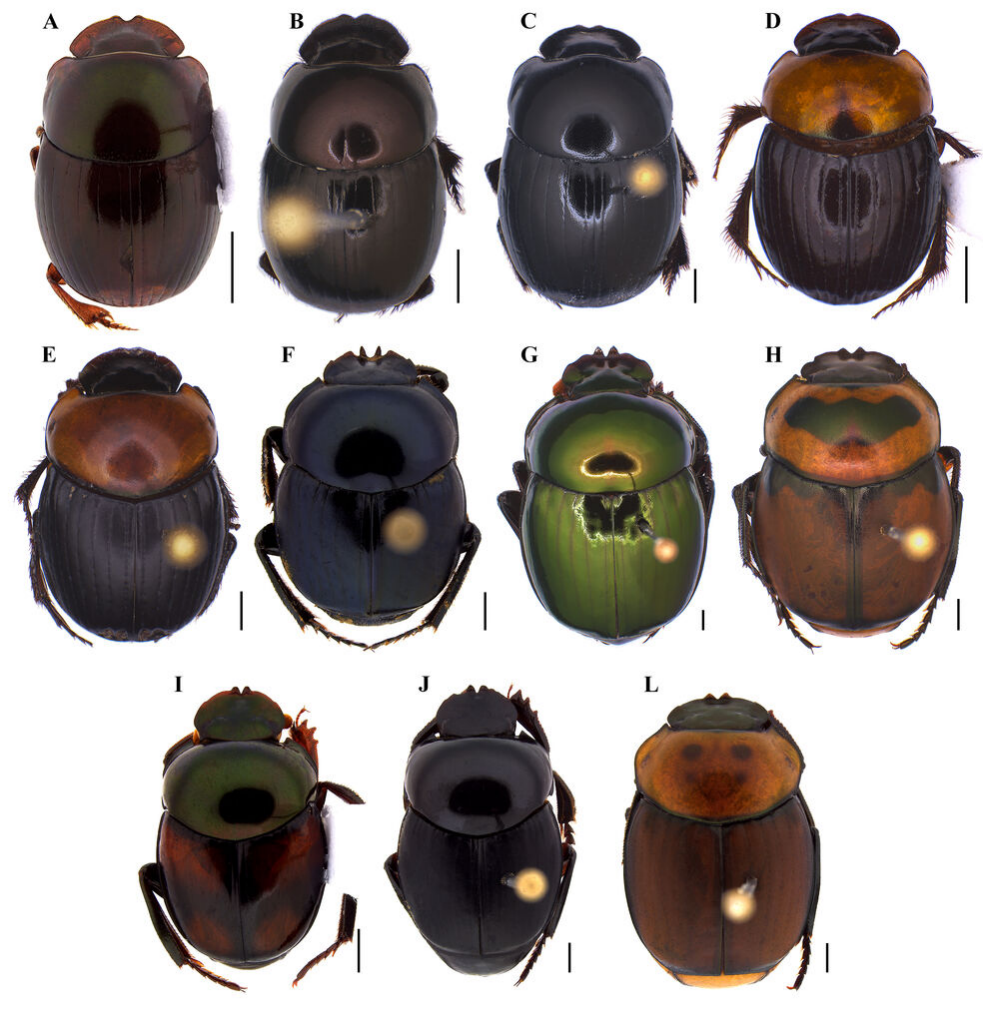
Dorsal habitus of dung beetle species collected in the Tapajós National Forest and/or Carajás National Forest **A** *Ateuchusglobulus* (Balthasar, 1938); **B** *Ateuchussemicupreus* (Harold, 1868); **C** *Ateuchussubstriatus* (Harold, 1868); **D** *Canthidiumdeyrollei* Harold, 1867; **E** *Canthidiummelanocephalum* (Olivier, 1789); **F** *Canthonconformis* Harold, 1868; **G** *Canthonfulgidus* Redtenbacher, 1868; **M** *Canthonhistrio* (Lepeletier de Saint Fargeau & Audinet-Serville, 1828); **I** *Canthonsubhyalinus* (Rivera-Cervants & Halffter, 1999); **J** *Canthonsemiopacus* Harold, 1868; **L** *Canthontriangularis* (Drury, 1770). Scale bar: 1 mm.

With around 100 species described, this genus needs urgent revision. The last revision of Brazilian *Ateuchus* species was done by Harold (1868), while Balthasar (1939) represents the last identification key for the genus. Most species occur in North America, Costa Rica and Mexico (Kohlmann 1984, Kohlmann 1997, Génier 2000, Kohlmann and Vaz-de-Mello 2018) and appear to be copro-necrophagous, including species from open areas and species that live associated with ant nests (Vaz-de-Mello et al. 1998). Nine species were identified in our study: *Ateuchusglobulus* (Balthasar, 1938), A.cf.pygidialis, A.cf.murrayi, *A.semicupreus* (Harold, 1868), *A.substriatus* (Harold, 1868) and *Ateuchus* sp. 1, *Ateuchus* sp. 2, *Ateuchus* sp. 3 and *Ateuchus* sp. 4 (Fig. 2A-G).

#### Canthidium

Erichson, 1847

D5028F6B-C019-5A1C-8458-1D7E8A2CC485

##### Notes

This is one of the most diverse dung beetle genera, comprising around 180 described species (Génier and Cupello 2018, Schoolmeesters 2022). Numerous species were described in small revisions, synopses and regional studies (e.g. Boucomont (1928), Balthasar (1939), Martínez et al. (1964), Howden and Young (1981), Solis and Kohlmann (2004), Kohlmann and Solís (2006)), while new species are expected to be described (Cupello 2018, Kohlmann et al. 2018, Santana et al. 2019). *Canthidium* species have been recorded within Neotropical forests and intra-Amazonian savannahs (e.g. França et al. (2016)). Although little is known about most species’ habits, specimens are easily collected in traps baited with faeces, rotten fruit and/or light traps (e.g. Medri and Lopes (2001), Silva and Audino (2011), Silva et al. (2014)), while some species were observed feeding on fungus (Falqueto et al. 2005). The specimens were identified from comparison with the original types and descriptions, which were analysed by one of the authors. Two species were identified to the species level in our survey in FLONA Tapajós: *Canthidiumdeyrollei* Harold, 1867 and *C.melanocephalum* (Olivier, 1789) (Fig. 2H-I). Other 22 species are present, but could not be identified.

#### Canthon

Hoffmannsegg, 1817

7B7AA07E-9088-5509-AB43-A6709216C080

##### Notes

This is also a very diverse genus, comprising more than 170 described species (Halffter and Martinez 1977). Most species are considered copro-necrophagous, although some exhibit predatory behaviour – for example, hunting ants (Halffter and Matthews 1966) – or use dead insects and millipedes (Villalobos et al. 1988, Silva et al. 2014), rotten fruits and fungus as food resources (Vaz-de-Mello 1999). This genus is endemic to the Americas and its distribution ranges from the USA to Uruguay and northern Argentina. Recent revisions have been made for some *Canthon* subgenera (Nunes et al. 2018, Nunes et al. 2020). Typically, these species are abundant in lowland forest environments, with individuals found perching on leaves exposed to light (Nunes et al. 2018). Another important point to be discussed is the population of *Canthonfulgidus* Redtenbacher, 1868 with green colour living in eastern Amazonia. According to Nunes et al. (2018), the population with green colour, named *Canthonfulgidusmartinezi* Nunes et al., 2018, is restricted to the western Amazon, while the populations from Carajás and Tapapós regions were expected to have a red metallic colour (named by the authors as *Canthonfulgiduspereirai* Nunes et al., 2018). This new finding (both green and red populations collected in the same region) suggests that Nunes et al. (2018) may have overlooked the green specimens from eastern Amazonia, as previously mentioned by Cupello et al. (2021), who discuss the colour variation and geographical distribution of distinct Scarabaeinae beetles. For the identification of species, the following works were mainly used: Nunes et al. (2018), Nunes et al. (2020). Nine species were identified: Canthonaff.histrio, C.aff.sericatus, C.aff.xanthopus, *C.conformis* Harold, 1868, *C.fulgidus* Redtenbacher, 1868, *C.histrio* (Lepeletier de Saint-Fargeau & Audinet-Serville, 1828), *C.subhyalinus* (Rivera-Cervants & Halffter, 1999), *C.semiopacus* Harold, 1868 and *C.triangularis* (Drury, 1770) (Fig. 2J-P).

#### Coprophanaeus

d’Olsoufieff, 1924

71914056-CBD6-5BB7-9F61-DCCE1A29667F

##### Notes

**Figure 3.**
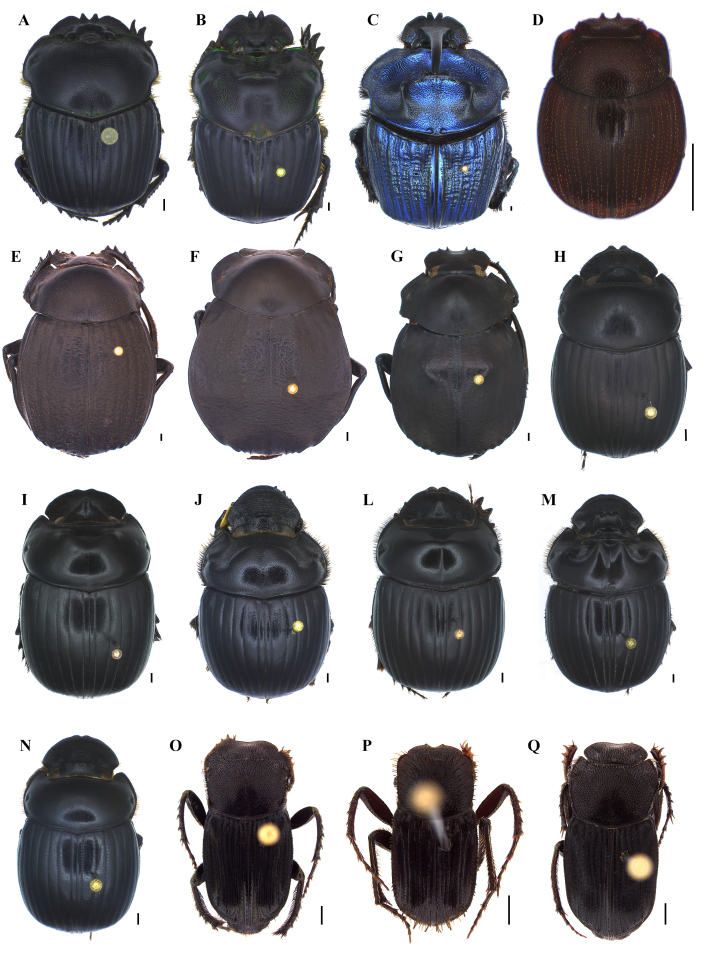
Dorsal habitus of dung beetle species collected in the Tapajós National Forest and/or Carajás National Forest. **A** *Coprophanaeusdegallieri* Arnaud, 1997; **B** *Coprophanaeusjasius* (Olivier, 1789); **C** *Coprophanaeuslancifer* (Linnaeus, 1767); **D** *Cryptocanthoncampbellorum* Howden, 1973; **E** *Deltochilumanceladus* Kolbe, 1893; **F** *Deltochilumorbiculare* Lansberge, 1874; **G** *Deltochilumorbignyiamazonicum* Bates, 1887; **H** *Dichotomiuscuprinus* (Felsche, 1901); **I** *Dichotomiusmamillatus* (Felsche, 1901); **J** *Dichotomiusmelzeri* (Luederwaldt, 1922); **L** *Dichotomiusnisus* (Olivier, 1789); **M** *Dichotomiuspelamon* (Harold, 1869); **N** *Dichotomiusworontzowi* (Pereira, 1942); **O** *Eurysternusarnaudi* Génier, 2009; **P** *Eurysternusatrosericus* Génier, 2009; **Q** *Eurysternusbalachowski* Halffter & Halffter, 1977. Scale bar: 1 mm.

A Neotropical genus with approximately 51 known species (Schoolmeesters 2022), which are easily identified using the taxonomic keys published by Edmonds and Zidek (2010). This genus is known to be attracted to carcasses and be captured in flight intercept traps (Vaz-de-Mello 1999). Usually found in fresh carrion at dusk periods (Halffter and Matthews 1966). For the identification of species, the following works were mainly used: Edmonds and Zidek (2010). Three species were identified in our study: *Coprophanaeusdegallieri* Arnaud, 1997, *C.jasius* (Olivier, 1789) and *C.lancifer* (Linnaeus, 1767) (Fig. 3A-C).

#### Cryptocanthon

Balthasar, 1942

84631DC2-55F5-596F-8AEA-8E9A7750DF1C

##### Notes

This genus comprises around 43 species occurring from Brazil to Mexico (Arias and Medina 2014, Martínez-Revelo et al. 2020, Giraldo-Mendonza 2022). The only available information about their habitat describes specimens inhabiting the leaf litter of humid and tropical forests, both in mountains and low altitudes (Cook 2002). For the identification of the species, the following works were mainly used: Cook (2002). *Cryptocanthoncampbellorum* Howden, 1973 (Fig. 3D) was the only species, which is usually collected in leaf litter, with flight interception and pitfall traps baited with human faeces (Cook 2002).

#### Deltochilum

Eschscholtz, 1822

9481482D-27D2-5F30-9322-0553AF209307

##### Notes

This is a very diverse genus of the Americas, with approximately 115 described species (González-Alvarado and Vaz-de-Mello 2021). *Deltochilum* individuals are mostly nocturnal and often found in temperate, tropical and subtropical forests (Halffter and Matthews 1966). *Deltohyboma* is currently the most challenging subgenus, which has been recently revised with several new species (González-Alvarado and Vaz-de-Mello 2021). For the identification of the species, the following works were mainly used: González-Alvarado and Vaz-de-Mello (2021). Seven species were identified through our study: *Deltochilumenceladus* Kolbe, 1893, *D.* gp. *aspericolle*, *D.* gp. *guyanense*, *D.* gp. *sextuberculatum*, *D.orbiculare* van Lansberge, 1874, *D.orbignyiamazonicum* Bates, 1887 and *Deltochilum* sp. 1 (Fig. 3E-G).

#### Dichotomius

Hope, 1838

62225191-C053-576D-B628-90A2BEC89D5B

##### Notes

According to the most recent taxonomic revision from one of the subgenera of *Dichotomius*, this Neotropical genus comprises around 190 species widely distributed from the USA to Argentina (Nunes and Vaz-de-Mello 2019). The four subgenera are either being revised or were recently revised (Nunes et al. 2016, Valois et al. 2017, Maldaner et al. 2018, Nunes and Vaz-de-Mello 2019). *Dichotomius* species occur in all Brazilian biomes and can be collected in pastures, savannahs or forests. The genus as a whole is considered paracoprid – i.e. tunnellers (Nunes and Vaz-de-Mello 2019). For the identification of the species, the following works were mainly used: Nunes et al. (2016), Valois et al. (2017), Maldaner et al. (2018), Nunes and Vaz-de-Mello (2019). We collected nine species: Dichotomiusaff.batesi, D.aff.lucasi 1, D.aff.lucasi 2, *D.cuprinus* (Felshe, 1901), *D.mamillatus* (Felshe, 1901), *D.melzeri* (Luederwaldt, 1922), *D.nisus* (Olivier, 1789), *D.pelamon* (Harold, 1869) and *D.worontzowi* (Pereira, 1942) (Fig. 3H-N).

#### Eurysternus

Dalman, 1824

1754C209-0766-555F-89C7-481186DE42C2

##### Notes

**Figure 4.**
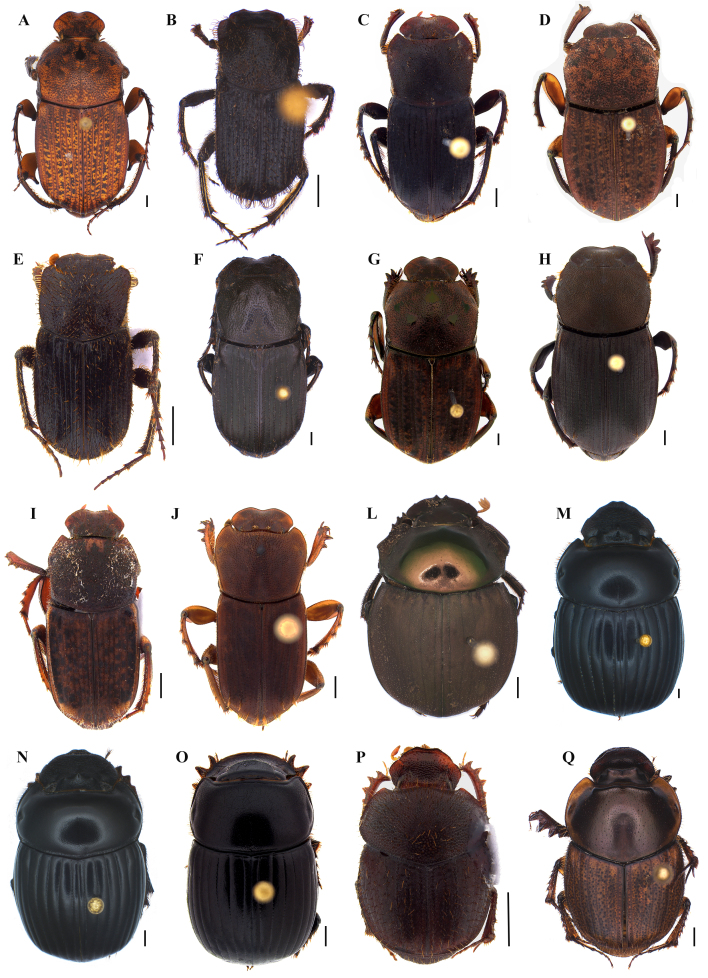
Dorsal habitus of dung beetle species collected in the Tapajós National Forest and/or Carajás National Forest. **A** *Eurysternuscaribaeus* (Herbst, 1789); **B** *Eurysternuscavatus* Génier, 2009; **C** *Eurysternuscayennensis* Castelnau, 1840; **D** *Eurysternuscyclops* Génier, 2009; **E** *Eurysternusfallaciosus* Génier, 2009; **F** *Eurysternusfoedus* Guérin-Méneville, 1844; **G** *Eurysternushamaticollis* Balthasar, 1939; **H** *Eurysternushypocrita* Balthasar, 1939; **I** *Eurysternusplebejus* Harold, 1880; **J** *Eurysternuswittmerorum* Martinez, 1988; **L** *Hansreiaoxygona* (Perty, 1830); **M** *Isocoprisimitator* (Felsche, 1901); **N** *Isocoprisnitidus* (Luederwaldt, 1922); **O** *Ontheruscarinifrons* Luederwaldt, 1930; **P** *Onthophagusdigitifer* Boucomont, 1932; **Q** *Onthophagusonthochromus* Arrow, 1913. Scale bar: 1 mm.

A Neotropical genus with 53 described species (Génier 2009) that are mostly endocoprids (Halffter and Matthews 1966, Cupello and Vaz-de-Mello 2018). *Eurysternus* species are easily collected in pitfalls baited with faeces, occurring in forests and frequently abundant in flooding-prone areas (Génier 2009). Génier (2009) was used for species identification. Thirteen species were recorded in our surveys: *Eurysternusarnaudi* Génier, 2009, *E.atrosericus* Génier, 2009, *E.balachowskyi* Halffter & Halffter, 1977, *E.caribaeus* (Herbst, 1789), *E.cavatus* Génier 2009, *E.cayannensis* Castelnau, 1840, *E.cyclops* Génier, 2009, *E.fallaciosus* Génier, 2009, *E.foedus* Guérin-Méneville, 1844, *E.hamaticollis* Balthasar, 1939, *E.hypocrita* Balthasar, 1939, *E.plebejus* Harold, 1880 and *E.wittmemorum* Martínez, 1988 (Fig. 3O-Q, Fig. 4A-J).

#### Eutrichilum

Martínez, 1969

616515D5-ABD4-595F-807A-5F94DDF709FD

##### Notes

This genus presents a group of species that inhabit South American lowlands, east of the Andes as far south as Buenos Aires in Argentina; one species in Costa Rica (Vaz-De-Mello 2008). Species of this genus are frequently necrophagous and are often attracted to light (Vaz-De-Mello 2008). *Eutrichillum* sp. 1 was the only species recorded within the Tapajós region.

#### Hansreia

Halffter & Martínez, 1977

8113E838-9910-533E-8985-544E7E8CFF6A

##### Notes

This is an Amazonian genus with six species distributed across Brazil, French Guiana and Venezuela (Halffter and Martinez 1977); recently revised by Valois et al. (2015). Valois et al. (2015) was used for species identification. There is not much ecological information about *Hansreia* dung beetles (Hadara et al. 2020). Only the species *Hansreiaoxygona* (Perty, 1830) was recorded within the Carajás region (Fig. 4L).

#### Isocopris

Pereira e Martínez, 1960

9C3F113F-DB8F-5B4D-A712-CCD8462290A5

##### Notes

This Neotropical genus, frequently misidentified as *Dichotomius*, comprises seven known species recently revised by Rossini and Vaz-de-Mello (2017), the same work being used to identify the species. No biological information for the genus was found. Two species were identified in our study: *Isocoprisimitator* (Felsche, 1901) and *I.nitidus* (Luederwaldt, 1922) (Fig. 4M-N).

#### Ontherus

Erichson, 1847

6CE01CB8-9814-557C-A3E9-47198EDC6936

##### Notes

Occurring from Argentina to Mexico, this genus has approximately 60 species (Génier 1996, Génier 1998). Although most species are considered coprophagous or saprophagous, some complex associations with ants have been previously recorded (Génier 1996). For the identification of the species, the following works were mainly used: Génier (1996). One species was found in the Tapajós region: *Ontheruscarinifrons* Luederwaldt, 1930 (Fig. 4O). This species belongs to a group called *appendiculatus*, which is widely distributed in South America (Génier 1996). Species from this group are usually collected in human or cattle dung, also using flight or light traps in sandy habitats (Génier 1996).

#### Onthophagus

Latreille, 1802

327C1801-9764-56B4-9639-48117B5C967E

##### Notes

**Figure 5.**
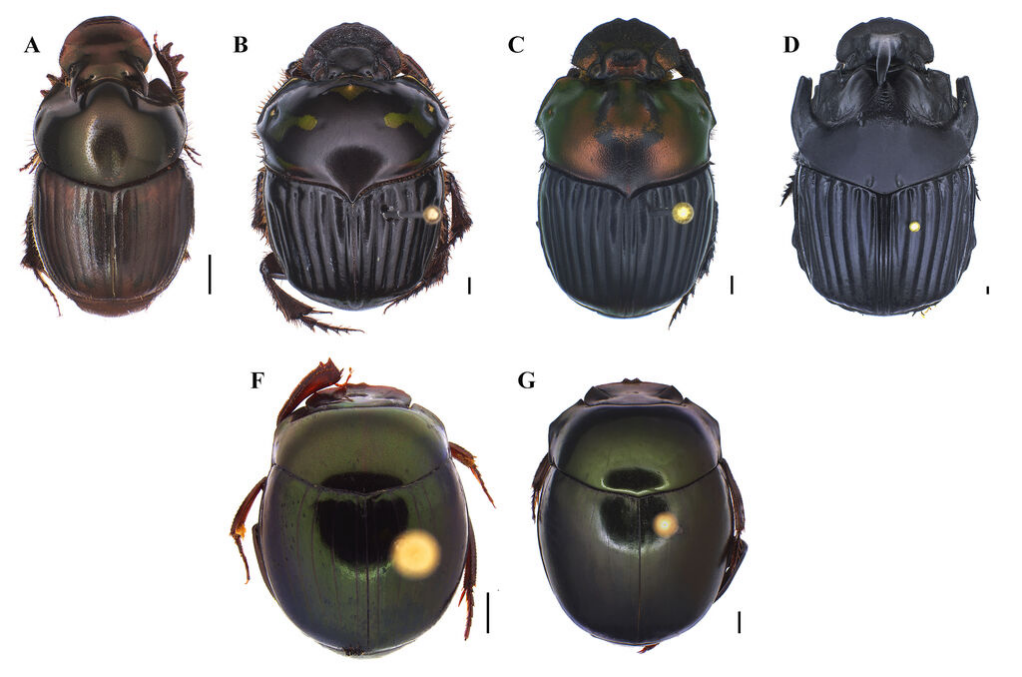
Dorsal habitus of dung beetle species collected in the Tapajós National Forest and/or Carajás National Forest. **A** *Onthophagusosculatii* Guérin-Méneville, 1855; **B** *Oxysternonmacleayi* Nevison, 1892; **C** *Oxysternonsilenus* Castelnau, 1840; **D** *Sulcophanaeusfaunus* (Fabricius, 1775); **E** *Silvycanthoncandezei* (Harold, 1869); **F** *Sylvicanthonproseni* (Martínez, 1949). Scale bar: 1 mm.

Considered a megadiverse and cosmopolitan genus with approximately 2,000 described species (Tarasov and Kabakov 2010). Some species have been recently revised (Rossini et al. 2018a, Rossini et al. 2018b), while others are under current revision. The species mentioned here have been recorded mainly in primary and secondary forests, through the use of both flight interception traps and pitfalls baited with dung or carrion (Korasaki et al. 2012). For the identification of the species, the following works were mainly used: Rossini et al. (2018a), Rossini et al. (2018b). Four species were identified in our study: *Onthophagusdigitifer* Boucomont, 1932, *O.* gp. *rubrescens*, *O.onthochromus* Arrow, 1913 and *O.osculatii* Guérin-Méneville, 1855 (Fig. 5A).

#### Oxysternon

Castelnau, 1840

7C9BC28E-4DF3-5BA4-8828-14CF9DCDDFA1

##### Notes

This Neotropical genus comprises 11 species according to the last taxonomic revision by Edmonds and Zídek (2004) which was used for species identification. *Oxysternon* beetles are usually found in primary and secondary forests (Gigliotti et al. 2011). The literature on the biology of the genus is scarce, but most species are considered as coprophagous and inhabit moist forests (Edmonds and Zídek 2004). Two species were recorded in this study: *Oxysternonmacleayi* Nevinson, 1892 and *O.silenus* Castelnau, 1840 (Fig. 5B-C).

#### Scybalocanthon

Martínez, 1948

09A80AAD-2A11-554C-89F3-049C48141D50

##### Notes

*Scybalocanthon* is a widespread genus occurring in South and Central America (Pereira and Martínez 1956, Silva and Valois 2019). The genus comprises 24 valid species, most of which are diurnal and inhabit either moist or dry forests in the Amazon Region, Atlantic Rainforest and the Yungas (Silva and Valois 2019, Silva and Génier 2019). Only the species *Scybalocanthon* sp. 1 was reported to the Carajás region.

#### Sulcophanaeus

d'Olsoufieff, 1924

F5D6B9A5-8B20-566E-B893-80FC69AE3EDE

##### Notes

This Neotropical genus has approximately 15 described species (Edmonds 2000). Morelli et al. (1996)Noriega (2001) bring information about the life cycle of some *Sulcophanaeus* species. *Edmonds (2000)* was used for species identification. *Sulcophanaeusfaunus* (Fabricius, 1775) was the only species recorded in this study (Fig. 5D).

#### Sylvicanthon

Halffter & Martínez, 1977

92A3E0D0-A170-5D42-A64B-41E2C4C1CF8C

##### Notes

This genus has 15 species, broadly occurring in the Neotropics and was recently revised by Cupello and Vaz-de-Mello (2018). Some species are widespread in the Amazon Basin (Cupello and Vaz-de-Mello 2018). All known species are nocturnal, with most of them considered coprophagous feeding on primate, pig and cattle dung (Cupello and Vaz-de-Mello 2018). For the identification of the species, the following works were mainly used: Cupello and Vaz-de-Mello (2018). Two species were identified in our study: *Sylvicanthoncandezei* (Harold, 1869) and *S.proseni* (Martínez, 1949) (Fig. 5E-F).

#### Uroxys

Westwood, 1842

7AC56DC3-CCDF-5381-9DAE-429382EEEC5D

##### Notes

*Uroxys* is an exclusively Neotropical genus with more than 50 described species (Vaz-De-Mello 2008, Korasaki et al. 2012). This genus has species that can be found in grasslands and within primary and secondary forests; it also includes species specialised in sloth (Bradypodidae) dung (Korasaki et al. 2012). Uroxyscf.minutus was the only species reported in this study.

## Discussion

The knowledge of biodiversity is key to providing information for conservation and management strategies, particularly within the hyperdiverse tropics (Barlow 2018). Our dung beetle surveys within FLONA Tapajós and Carajás highlight the importance of Amazonian Sustainable-Use protected forests for conserving insect biodiversity in the tropics. Protected areas have a key role as a thermal buffer against climate changes (Xu et al. 2022) and for the long-term maintenance of Amazonian biodiversity (Laurance 2005). However, Brazilian protected forests are largely underfunded, particularly in Amazonia (Silva et al. 2021) – which hinders their efficacy in protecting biodiversity and raises the urgency for new policies and funding mechanisms to enhance their efficacy.

## Supplementary Material

XML Treatment for Scarabaeinae

XML Treatment for Anomiopus

XML Treatment for Ateuchus

XML Treatment for Canthidium

XML Treatment for Canthon

XML Treatment for Coprophanaeus

XML Treatment for Cryptocanthon

XML Treatment for Deltochilum

XML Treatment for Dichotomius

XML Treatment for Eurysternus

XML Treatment for Eutrichilum

XML Treatment for Hansreia

XML Treatment for Isocopris

XML Treatment for Ontherus

XML Treatment for Onthophagus

XML Treatment for Oxysternon

XML Treatment for Scybalocanthon

XML Treatment for Sulcophanaeus

XML Treatment for Sylvicanthon

XML Treatment for Uroxys

## Figures and Tables

**Table 1.** List and abundance of species present in FLONA Tapajós and Carajás.

**Species**	**Abundance**	
	**FLONA Tapajós**	**FLONA Carajás**
Anomiopusaff.pereirai	1	-
*Anomiopus* sp. 2	1	-
*Anomiopus* sp. 3	1	-
*Anomiopus* sp. 4	1	-
*Anomiopus* sp. 5	-	1
*Ateuchusglobulus* (Balthasar, 1938)	1	-
*Ateuchus* sp. 2	14	-
Ateuchus sp. 3	10	-
Ateuchus gp. pygidialis	-	1
Ateuchuscf.murrayi	113	-
*Ateuchus* sp. 4	64	-
*Ateuchussemicupreus* (Harold, 1868)	-	18
*Ateuchus* sp. 1	-	39
*Ateuchussubstriatus* (Harold, 1868)	12	-
*Canthidiumdeyrollei* Harold, 1867	283	-
*Canthidiummelanocephalum* (Olivier, 1789)	31	-
*Canthidium* sp. 1	-	13
*Canthidium* sp. 2	1	-
*Canthidium* sp. 3	-	3
*Canthidium* sp. 4	-	29
*Canthidium* sp. 5	84	-
*Canthidium* sp. 6	6	-
*Canthidium* sp. 7	6	-
*Canthidium* sp. 9	1	-
*Canthidium* sp. 10	86	-
*Canthidium* sp. 11	4	-
*Canthidium* sp. 12	5	-
*Canthidium* sp. 13	-	2
*Canthidium* sp. 14	-	346
*Canthidium* sp. 15	128	-
*Canthidium* sp. 18	15	-
*Canthidium* sp. 19	3	-
*Canthidium* sp. 20	1	-
*Canthidium* sp. 21	1	-
*Canthidium* sp. 22	-	2
*Canthidium* sp. 25	-	4
*Canthidium* sp. 26	-	1
*Canthidium* sp. 33	-	3
Canthonaff.histrio	34	-
Canthonaff.sericatus	1	-
Canthonaff.xanthopus	9	-
*Canthonconformis* Harold, 1868	1	-
*Canthonfulgidus* Redtenbacher, 1868	235	29
*Canthonhistrio* (Lepeletier de Saint-Fargeau & Audinet-Serville, 1828)	-	35
*Canthonsubhyalinus* (Rivera-Cervants & Halffter, 1999)	2	-
*Canthonsemiopacus* Harold, 1868	6	0
*Canthontriangularis* (Drury, 1770)	-	3
*Coprophanaeusdegallieri* Arnaud, 1997	1	-
*Coprophanaeusjasius* (Olivier, 1789)	3	-
*Coprophanaeuslancifer* (Linnaeus, 1767)	27	18
*Cryptocanthoncampbellorum* Howden, 1973	4	3
*Deltochilumenceladus* Kolbe, 1893	4	-
*Deltochilum* gp. *aspericolle*	156	-
*Deltochilum* gp. *guyanense*	13	45
*Deltochilum* gp. *sextuberculatum*	6	-
*Deltochilumorbiculare* Van Lansberge, 1874	2	27
*Deltochilumorbignyiamazonicum* Bates, 1887	5	-
*Deltochilum* sp. 1	3	-
Dichotomiusaff.batesi	225	36
Dichotomiusaff.lucasi 1	137	50
Dichotomiusaff.lucasi 2	112	-
*Dichotomiuscuprinus* (Felshe, 1901)	-	1
*Dichotomiusmamillatus* (Felshe, 1901)	-	5
*Dichotomiusmelzeri* (Luederwaldt, 1922)	2	-
*Dichotomiusnisus* (Olivier, 1789)	-	51
*Dichotomiuspelamon* (Harold, 1869)	5	1
*Dichotomiusworontzowi* (Pereira, 1942)	3	2
*Eurysternusarnaudi* Génier, 2009	8	-
*Eurysternusatrosericus* Génier, 2009	192	-
*Eurysternusbalachowskyi* Halffter & Halffter, 1977	4	-
*Eurysternuscaribaeus* (Herbst, 1789)	163	86
*Eurysternuscavatus* Génier, 2009	-	3
*Eurysternuscayennensis* Castelnau, 1840	10	-
*Eurysternuscyclops* Génier, 2009	-	1
*Eurysternusfallaciosus* Génier, 2009	-	2
*Eurysternusfoedus* Guérin-Méneville, 1844	-	12
*Eurysternushamaticollis* Balthasar, 1939	2	1
*Eurysternushypocrita* Balthasar, 1939	1	-
*Eurysternusplebejus* Harold, 1880	5	-
*Eurysternuswittmerorum* Martínez, 1988	47	44
*Eutrichillum* sp. 1	1	-
*Hansreiaoxygona* (Perty, 1830)	-	17
*Isocoprisimitator* (Felsche, 1901)	3	-
*Isocoprisnitidus* (Luederwaldt 1922)	2	-
*Ontheruscarinifrons* Luederwaldt, 1930	13	-
*Onthophagusdigitifer* Boucomont, 1932	1	-
*Onthophagus* gp. rubrescens	91	147
*Onthophagusonthochromus* Arrow, 1913	-	1
*Onthophagusosculatii* Guérin-Méneville, 1855	70	10
*Oxysternonmacleayi* Nevinson, 1892	26	11
*Oxysternonsilenus* Castelnau, 1840	1	3
*Scybalocanthon* sp. 1	-	2
*Sulcophanaeusfaunus* (Fabricius, 1775)	1	-
*Sylvicanthoncandezei* (Harold, 1869)	1	-
*Sylvicanthonproseni* (Martínez, 1949)	116	-
Uroxyscf.minutus	29	8
